# CDK8/19 inhibition plays an important role in pancreatic β-cell induction from human iPSCs

**DOI:** 10.1186/s13287-022-03220-4

**Published:** 2023-01-05

**Authors:** Kensuke Sakuma, Noriko Tsubooka-Yamazoe, Kiyohiro Hashimoto, Nozomu Sakai, Shinya Asano, Saori Watanabe-Matsumoto, Takeshi Watanabe, Bunnai Saito, Hirokazu Matsumoto, Hikaru Ueno, Ryo Ito, Taro Toyoda

**Affiliations:** 1 iPSC-Derived Pancreatic Islet Cell (iPIC) Therapy Department, Orizuru Therapeutics Inc., Fujisawa, Kanagawa 251-8555 Japan; 2 Takeda-CiRA Joint Program for iPS Cell Applications (T-CiRA), Fujisawa, Kanagawa 251-8555 Japan; 3 Drug Safety Research and Evaluation Group, Takeda Pharmaceutical Company Limited, Kanagawa, 251-8555 Japan; 4 Drug Discovery Sciences, Takeda Pharmaceutical Company Limited, Kanagawa, 251-8555 Japan; 5 Integrated & Translational Science, Axcelead Drug Discovery Partners, Inc., Fujisawa, Kanagawa 251-8555 Japan; 6 T-CiRA Discovery and Innovation, Takeda Pharmaceutical Company Limited, Kanagawa, 251-8555 Japan; 7 Department of Life Science Frontiers, Center for iPS Cell Research and Application (CiRA), Kyoto University, Kyoto, 606-8507 Japan

**Keywords:** Human-induced pluripotent stem cells, Mutagenicity, Pancreatic islet cell, Activin receptor-like kinase 5 inhibitor II, CDK8/19 inhibitors

## Abstract

**Background:**

Transplantation of differentiated cells from human-induced pluripotent stem cells (hiPSCs) holds great promise for clinical treatments. Eliminating the risk factor of malignant cell transformation is essential for ensuring the safety of such cells. This study was aimed at assessing and mitigating mutagenicity that may arise during the cell culture process in the protocol of pancreatic islet cell (iPIC) differentiation from hiPSCs.

**Methods:**

We evaluated the mutagenicity of differentiation factors used for hiPSC-derived pancreatic islet-like cells (iPICs). We employed Ames mutagenicity assay, flow cytometry analysis, immunostaining, time-resolved fluorescence resonance energy transfer-based (TR-FRET) cell-free dose–response assays, single-cell RNA-sequencing and in vivo efficacy study.

**Results:**

We observed a mutagenic effect of activin receptor-like kinase 5 inhibitor II (ALK5iII). ALK5iII is a widely used β-cell inducer but no other tested ALK5 inhibitors induced β-cells. We obtained kinase inhibition profiles and found that only ALK5iII inhibited cyclin-dependent kinases 8 and 19 (CDK8/19) among all ALK5 inhibitors tested. Consistently, CDK8/19 inhibitors efficiently induced β-cells in the absence of ALK5iII. A combination treatment with non-mutagenic ALK5 inhibitor SB431542 and CDK8/19 inhibitor senexin B afforded generation of iPICs with in vitro cellular composition and in vivo efficacy comparable to those observed with ALK5iII.

**Conclusion:**

Our findings suggest a new risk mitigation approach for cell therapy and advance our understanding of the β-cell differentiation mechanism.

**Supplementary Information:**

The online version contains supplementary material available at 10.1186/s13287-022-03220-4.

## Background

Type 1 diabetes is an autoimmune disease characterized by progressive pancreatic β-cell destruction and insulin deficiency, leading to severe hyperglycemia and various complications. Current standard care is exogenous insulin administration using advanced technologies [1]. In contrast, for patients with severe, “brittle”-type diabetes, who suffer from labile glycemic control and hypoglycemic events despite appropriate insulin treatment [2, 3], pancreas or islet transplantations are considered, but these interventions are hampered by the paucity of suitable organs for transplantation from deceased donors [2, 3]. Therefore, unlimited cell sources, such as human pluripotent stem cells (hPSCs), are critically needed [2–4].

Various multistep protocols have been described to generate hPSC-derived pancreatic cells in basic and translational medical research [5–10]. Small-molecule compounds targeting a specific protein or pathway were integrated into the protocols based on the developmental biology knowledge or unbiased screening results [11, 12]. Although such compounds would not be directly administered to patients, cells manufactured using these compounds could eventually be introduced into patients. Therefore, it would be preferable for these compounds to have a good safety profile, because genotoxic agents disturb genomic integrity, as do radiation and genome-integrating viruses, occasionally causing mutagenesis [13]. Supplementation with genotoxic compounds not only affects cell differentiation but also increases the mutagenicity risk at each step of the differentiation from hPSCs, including proliferative progenitor stages [14].

The purpose of this study was to assess and mitigate mutagenicity that may arise during the cell culture process in the protocol of pancreatic islet cell (iPIC) differentiation from human-induced pluripotent stem cells (hiPSCs) [15, 16].The majority of chemical compounds used by us are also frequently used in other protocols, targeting pancreatic cells and different cell types. Surprisingly, using the Ames test, we found the mutagenic potential of transforming growth factor-β (TGF-β) type I receptor kinase/activin receptor-like kinase 5 (TGF-βRI/ALK5) inhibitor II (ALK5iII), a widely used compound to induce pancreatic β-cells and other cell types, such as neurons and osteoblasts [17, 18]. Following the failure of screening for alternative ALK5 inhibitors, we explored off-target inhibition of kinases by ALK5iII and found that CDK8/19 inhibition is apparently a critical mechanism for efficient β-cell induction. Having compared the in vitro profiles of iPICs obtained following treatment with another ALK5 inhibitor and/or a CDK8/19 inhibitor, we proposed a non-mutagenic alternative that produced iPICs with in vivo efficacy comparable to that of iPICs generated using ALK5iII.

## Methods

### Cell culture and in vitro differentiation of hiPSCs

The human iPSC line Ff-I14-s04 was kindly provided by the Center for iPS Cell Research and Application (CiRA) at the Kyoto University. The Ff-I14s04 line was established as an HLA homozygous iPSC line in a previous study [19]. The use of the iPSC line was approved by the Ethical Review Committee of the Shonan Health Innovation Park (Fujisawa, Kanagawa, Japan) and Kyoto University (#CiRA18-27). Cells were cultured and maintained on dishes coated by iMatrix-511 (Nippi, Tokyo, Japan) in StemFit AK03N (Ajinomoto, Tokyo, Japan) at 37 °C in a humidified 5% CO_2_ incubator. Cells were passaged every 3 or 4 days by non-enzymatic dissociation, using 0.5 mM EDTA (Thermo Fisher Scientific, Waltham, MA, USA), and subjected to differentiation experiments usually after over a 2-week running culture.

For differentiation culture to generate iPICs, we used 2D monolayer to static aggregate culture based on our previous report [15] and 3D stirred-floating aggregate culture [16]. The details of the typical 3D floating culture were as follows. A special note here is that 10 μM ALK5 inhibitor II (ALK5iII, Santa Cruz) at Stages 5–7 is replaced with ALK5 inhibitors, CDK8/19 inhibitors, and their combination, as alternatives to ALK5iII, depending on the purposes of Figs. 2, 3, 4 and 5.

### Stage 1

Dissociated undifferentiated iPSCs were resuspended at a density of 2 × 10^5^ cells/mL in the AK03N medium containing 10 μM Y-27632 (FUJIFILM Wako, Tokyo, Japan). The cells were cultured in a spinner type 30 mL bioreactor (ABLE Corporation & Biott Co., Ltd., Tokyo, Japan), or a vertical mixing 0.25 L bioreactor (SATAKE MultiMix Corporation, Saitama, Japan) throughout culturing. The next day, the aggregated cells were cultured in DMEM medium (Thermo Fisher Scientific) supplemented with 1% (v/v) penicillin/streptomycin (P/S, FUJIFILM Wako), 1× B27 (#17504001 or A1895601; Thermo Fisher Scientific), 1% *Pluronic*® F-68 (Sigma-Aldrich Co. LLC, Saint Louis, MO, USA) to reduce the fluid-induced mechanical damage, 5–10 ng/mL activin A (PeproTech, Cranbury, NJ, USA), 3 μM CHIR99021 (Axon Medchem, Reston, VA, USA), and 1% DMSO (FUJIFILM Wako). The following day, CHIR99021 was removed from the medium, and the culture was continued for another 2 days.

### Stage 2

Cells were cultured in the MCDB 131 Medium (Thermo Fisher Scientific) supplemented with 1% P/S, 0.5× B27, 1% *Pluronic*® F-68, 50 ng/mL keratinocyte growth factor (KGF, R&D Systems, Minneapolis, MN, USA), 4.44 mM glucose (final concentration 10 mM, FUJIFILM Wako), 1.5 g/L NaHCO_3_ (FUJIFILM Wako), and 1% GlutaMAX (Thermo Fisher Scientific) for 4 days.

### Stage 3

Cells were cultured in the improved MEM (iMEM, Thermo Fisher Scientific) containing 1% P/S, 0.5× B27, 1% *Pluronic*® F-68, 50 ng/ml KGF, 100 ng/mL Noggin (FUJIFILM Wako), 0.5 μM 3-keto-N-aminoethyl-N′-aminocaproyldihydrocinnamoyl cyclopamine (KAAD-cyclopamine, Toronto Research Chemicals, Toronto, Canada), and 10 nM 4-[(E)-2-(5,6,7,8-tetrahydro-5,5,8,8-tetramethyl-2-naphthalenyl)-1-propenyl] benzoic acid (TTNPB, Santa Cruz Biotechnology, CA, USA) for 3 days.

### Stage 4

Cells were cultured in iMEM containing 1% P/S, 0.5× B27, 1% *Pluronic*® F-68, 100 ng/mL KGF, 50 ng/mL epidermal growth factor (EGF, R&D Systems), 10 mM nicotinamide (STEMCELL Technologies, Vancouver, Canada), 0.1 μM TR05991851 (ROCK inhibitor, Takeda Pharmaceutical Company, Osaka, Japan), 0.5 μM PDBu (Merck Millipore, Billerica, MA, USA), and 5 ng/mL activin A for 4 days.

In the following stages 5–7, we used 10 μM ALK5iII and their alternatives (ALK5 inhibitors, CDK8/19 inhibitors, and their combination such as 3 μM SB 431542 and 0.3 μM senexin B, depending on the experimental purposes as mentioned above.

### Stage 5

Cells were cultured in iMEM with 1% P/S, 0.5× B27, 1% *Pluronic*® F-68, 0.25 μM SANT-1 (Merck Millipore), 50 nM retinoic acid (Merck Millipore), 10 μM ALK5 inhibitor II (ALK5iII, Santa Cruz) or the alternative candidates, 100 nM LDN-193189 (MedChemExpress, Monmouth Junction, NJ, USA), 1 μM L-3,3′,5-triiodothyronine (T_3_, Merck Millipore), 50 ng/mL basic fibroblast growth factor (bFGF, PeproTech), 1 μM XAV939 (Merck Millipore), and 10 μM Y-27632 for 2 days.

### Stage 6

**Fig. 1:**
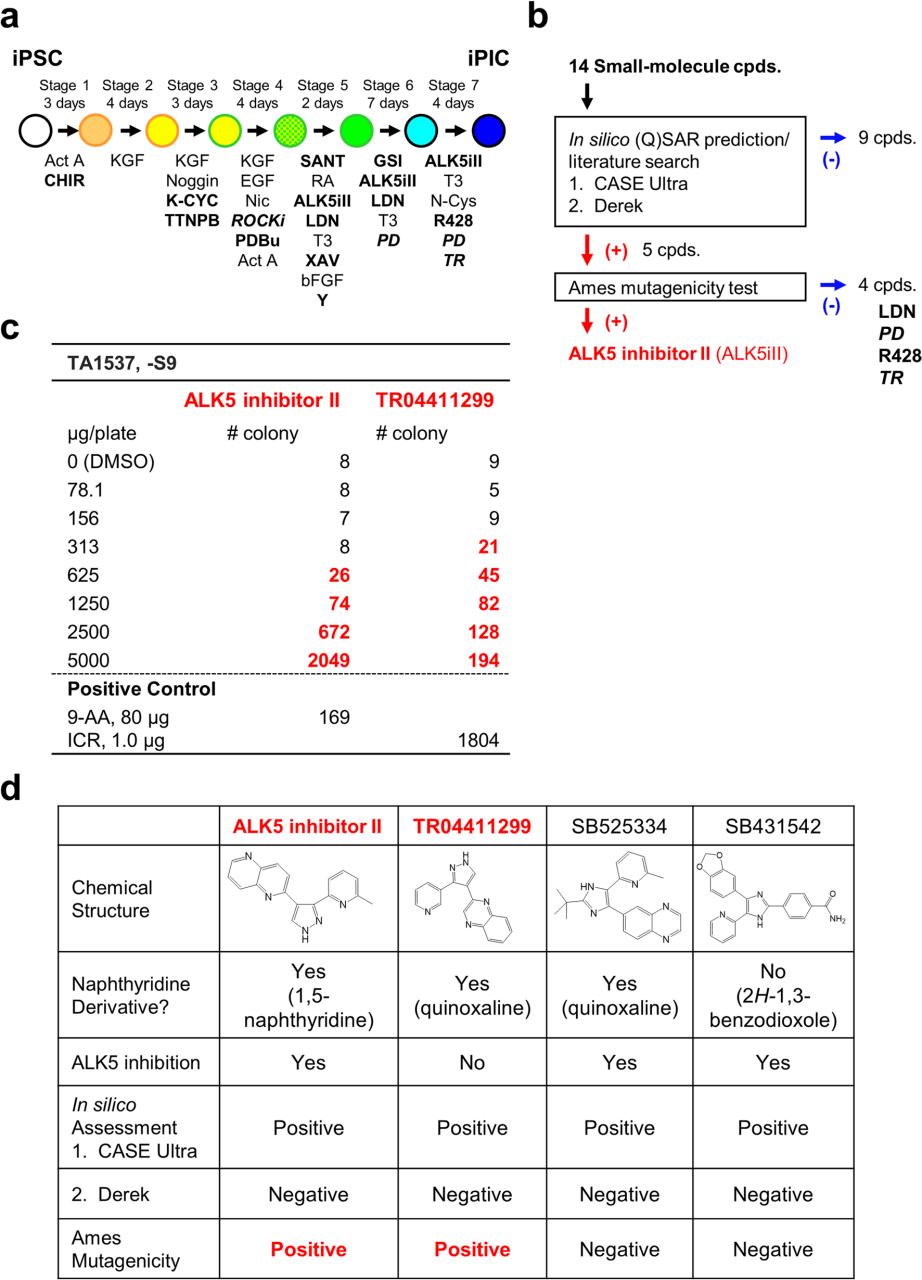
Mutagenicity of the naphthyridine derivative and endocrine cell inducer ALK5 inhibitor II in the Ames test.** a** Schematic representation of the protocol for iPIC differentiation from hiPSCs. Small-molecule compounds (14 reagents; 11 widely used reagents plus 3 original ones in italic) are highlighted in bold. Act A, activin A; CHIR, CHIR99021; K-CYC, KAAD-cyclopamine; TTNPB, 4-[(E)-2-(5,6,7,8-tetrahydro-5,5,8,8-tetramethyl-2-naphthalenyl)-1-propenyl] benzoic acid; *ROCKi, *TR05991851; PDBu, phorbol 12, 13-dibutyrate; SANT, SANT-1; ALK5iII, ALK5 inhibitor II; LDN, LDN-193189; XAV, XAV939; Y, Y-27632; GSI, RO4929097; *PD,* PD-166866; R428, bemcentinib; and *TR,* TR06141363. **b** Flowchart of mutagenicity evaluation of the 14 compounds used in the iPIC differentiation protocol. Red and blue arrows indicate positive and negative results, respectively. Cpds, compounds. **c** Positive results (more than twofold increase above the value in the concurrent vehicle control) in the bacterial reverse mutation assay in the absence of rat liver S9 fraction. 9-AA, 9-aminoacridine; ICR, ICR 191. **d** Summary of the mutagenicity evaluation results for ALK5iII, a non-ALK5 inhibitor structurally related to ALK5iII (TR04411299), and other ALK5 inhibitors (SB525334 and SB431542)

Cells were cultured in iMEM containing 1% P/S, 0.5× B27, 1% *Pluronic*® F-68, 1 μM RO4929097 (“GSI” in Fig. 1a, Chem Scene, Monmouth Junction, NJ, USA), 10 μM ALK5iII or the alternative candidates, 100 nM LDN-193189, and 1 μM T_3_ for 7 days. PD-166866 (1 μM, Merck Millipore) was added from day 4 of stage 6.

### Stage 7

Stage 7 medium was based on a previous report [7] with our original modifications. Cells were cultured in the MCDB 131 medium with 1% P/S, 2% fat-free bovine serum albumin (FUJIFILM Wako), 20 mM glucose, 1.5 g/L NaHCO_3_, 1% GlutaMAX, 0.5% ITS-X (Thermo Fisher Scientific), 10 μM ALK5iII or the alternative candidates, 1 μM T_3_, 10 μM ZnSO_4_ (Merck Millipore), 1.4 IU/mL heparin sodium salt (Nacalai Tesque, Kyoto, Japan), 1 mM N-acetyl cysteine (Merck Millipore), 10 μM Trolox (FUJIFILM Wako), 2 μM R428 (Selleck Chemicals, Houston, TX, USA), 1 μM PD-166866, 3 μM TR06141363 (Multi-kinase inhibitor, Takeda Pharmaceutical Company), and 10 μM Y-27632, for 4 days. To generate iPICs for implantation, cells were dissociated and re-sized in an Elplasia 6-well microwell plate (Corning Incorporated, Corning, NY, USA) or a gas-permeable microwell culture bag (Toyo Seikan Group Holdings, Ltd., Yokohama, Japan) [20] and cultured in the stage 7 medium.

### Ames mutagenicity assay

The Ames assay was performed to assess the mutagenicity of five compounds that displayed positive structural alerts in studies in silico (Fig. 1b). *Salmonella typhimurium* TA100, TA1535, TA98, and TA1537, and *Escherichia coli* WP2*uvrA* were used to detect either frameshift mutations (TA98 and TA1537) or base pair substitutions (TA100, TA1535, and WP2*uvrA*). These strains are widely used and recommended for use in ICH S2(R1) for the bacterial reversion assay.

In the current study, rat liver S9 fraction, an exogenous activator of post-mitochondrial supernatant, was not included because the mutagenicity of compounds should be assessed when simulating cell cultures but not in vivo after metabolism by liver enzymes. Briefly, each tester strain was mixed with seven concentrations (78.1–5000 μg/plate) of each compound and preincubated for 20 min in test tubes. Two plates per dose for the tested compounds and positive control group, and four plates per dose for the negative control group were used. Dimethyl sulfoxide (FUJIFILM Wako) was selected as solvent for the tested compounds and used as negative control. Strong mutagens served as positive controls: 9-aminoacridine hydrochloride monohydrate (9-AA, Sigma-Aldrich), and [6-Chloro-9-(3-[2-chloroethylamino]propylamino)-2-methoxyacridine] dihydrochloride (ICR 191, FUJIFILM Wako). Following preincubation, semisolid top agar was added to the tubes, and then, the mixtures were overlaid on minimal glucose agar plates. After the overlaid agar solidified, the plates were stored at 37 °C in an incubator for 48 h.

Cultures were examined for signs of compound precipitation and other abnormal conditions by eye. Subsequently, cytotoxicity (decreased background lawn) was assessed using a stereomicroscope. The number of revertant colonies on the plates was counted using an automatic colony analyzer (CA-11, System Science Co., Ltd., Tokyo, Japan) or manually when counting by the automatic colony analyzer was inappropriate, *e.g.*, when precipitation was observed. A positive response was defined as the mean number of revertant colonies that was at least twofold greater than the mean negative control value in any test strain.

### Flow cytometry analysis

The differentiation efficiency of β-cells (INS^+^NKX6.1^+^) and endocrine cells (CHGA^+^) was analyzed using immunostaining methods and flow cytometry on an LSRFortessa X20 instrument (BD Corporation, Franklin Lakes, NJ, USA), as described previously [5]. The primary antibodies used are detailed in Additional file 1: Table S1. Secondary antibodies were conjugated to Alexa Fluor 488, 546, 568, and 647 of the appropriate species (Thermo Fisher Scientific or Jackson ImmunoResearch, West Grove, PA, USA).

### Study of iPIC implantation in type 1 diabetic mice

Male immunodeficient NOD.CB17*-Prkdc*^*scid*^/J (NOD-*scid*) mice were purchased from Charles River Laboratories Japan (Yokohama, Japan). They were fed commercial diet CE-2 (CLEA Japan, Tokyo, Japan) and received tap water ad libitum, together with appropriate weekly sanitation and enrichment*.* The care and use of the animals as well as the experimental protocols in this study were approved by the Institutional Animal Care and Use Committee of the Shonan Health Innovation Park (Shonan iPark), Takeda Pharmaceutical Company. Animals were excluded if monitored health condition was severe in many aspects like weight, intake, activity, fur condition, and so on. For euthanasia of diabetic mice, for example, we use 50% CO_2_ at a flow rate of 5.0 L/min to 9.0 L M-2 chamber (CLEA Japan, Tokyo, Japan) for over minimally 10 min. We can increase the flow rates once animals have lost consciousness.

The experimental unit is the individual animal (*i.e.*, an implanted mouse) in this study. Male mice aged 8–9 weeks were intraperitoneally injected with five daily injections of streptozotocin (STZ, 50 mg/kg/day, Sigma-Aldrich) to induce type 1 diabetes and transferred to single housing to reduce fight, flight, freeze, or groom stress response that affect glucose levels. The hyperglycemic mice (n = 4–5 per group, total 14 mice for Fig. 5) were randomized based on blood glucose and body weight and were subcutaneously implanted with iPICs (3 × 10^6^ cells/mouse, implanted by a well-trained operator using a standardized protocol) embedded in fibrin gel obtained by mixing 10 mg/mL fibrinogen (Merck Millipore) and 50 IU/mL thrombin solutions (Sigma-Aldrich). Non-fasted blood glucose and plasma human C-peptide levels were measured every 2 or 4 weeks using ACCU-CHEK Aviva (Roche Diagnostics, Basel, Switzerland) and Ultrasensitive human C-peptide ELISA (Mercodia AB, Uppsala, Sweden), respectively, according to the manufacturers’ instructions. An oral glucose tolerance test was conducted 21 weeks after iPIC implantation. The plasma samples were obtained before and after the glucose loading (2 g/kg) at the indicated time points for the measurements of plasma glucose (Glucose autokit, 439–90901, FUJIFILM Wako) and human C-peptide levels. These measurements are generally conducted in type 1 diabetes pre-clinical studies [5–10].

### Immunostaining

For in vivo graft samples, the mice were dissected to obtain iPIC grafts for histological analysis over 6 months after the implantation. Paraformaldehyde (4%)-fixed grafts were dehydrated and embedded in paraffin. For in vitro aggregate samples, aggregates were frozen in Tissue-Tek O.C.T. compound (Sakura Finetek Japan, Tokyo, Japan) in a similar manner. Paraffin and frozen blocks were sectioned at 5–10 μm thickness, and they were used for immunofluorescence staining. After treatment with blocking solution (5% normal donkey serum/0.3% Triton X), the primary (Additional file 1: Table S1) and secondary antibodies (conjugated to Alexa Fluor 488 and 546 (Thermo Fisher Scientific)) were applied with appropriate wash steps. The sections were counterstained with Hoechst 33342 (1:200, Thermo Fisher Scientific) to label the nuclei. The sections were imaged using a fluorescent microscope (BZ-X700; Keyence, Osaka, Japan).

### TR-FRET-based kinase profiling assays for tested compounds

TR-FRET-based competitive binding assays for ~ 350 kinases have been developed and conducted by Axcelead Drug Discovery Partners (Fujisawa, Japan) [21]. Briefly, BODIPY-FL- or Cy5-FL-conjugated staurosporine derivative that binds to a highly conserved ATP-binding pocket in kinases was used as a fluorescent probe and tested compounds were applied at 0.1 and 1 μM in competitive binding assays for ~ 350 recombinant protein kinases. TR-FRET fluorescent signals from terbium and BODIPY/Cy5-FL were measured using an EnVision plate reader (PerkinElmer, Waltham, MA, USA), and pIC_50_ values were extrapolated from the inhibition rate.

### Single-cell RNA-sequencing and data processing

Single-cell RNA-seq libraries for iPICs were generated using the 10 × Genomics Chromium™ controller and Chromium Single Cell 3′ kits v3.1 (10 × Genomics, Pleasanton, CA, USA) according to the manufacturer’s instructions. Quality control and quantification of the obtained cDNA and library were conducted using high sensitivity DNA kits on an Agilent 2100 Bioanalyzer (Agilent Technologies, Palo Alto, CA, USA). The libraries were subjected to next-generation sequencing using the HiSeq platform (Illumina, San Diego, CA, USA) with 150 bp paired-end reads at a depth of > 100,000 reads per cell. Sequencing reads were aligned to the human GRCh38 genome reference, and gene counts were quantified as UMIs using Cell Ranger v5.0.1 (10 × Genomics). We imported UMI count matrices into R v3.6.3 software (Seurat v3.2.3 package) [22, 23] and performed normalization according to the package’s default setting. Cells with a percentage of mitochondrial gene counts over 20% were regarded as dead or damaged cells and removed from further analyses. UMI count matrices were scaled by regressing out the number of total UMI counts per cell and percentage of mitochondrial gene counts. Genes for dimensional reduction were selected based on the average expression and dispersion of each gene, and principal component analysis was performed. Principal components were used for Seurat’s shared nearest neighbor graph clustering, and uniform manifold approximation and projection dimensional reduction [24, 25] were used to visualize the data. Analysis of DEGs in each cluster was performed using the Wilcoxon rank-sum test in Seurat. Subsets of DEGs with fold change > 2 at *P* < 0.05 were extracted and applied to the publicly available database, PanglaoDB (https://panglaodb.se/) to identify cell-type labels using clusterProfiler v3.14.3 package [26].

### Statistical analysis

The Dunnett’s multiple comparison test was performed at a significance level of *P* < 0.05 to analyze the statistical significance of effects in iPIC differentiation studies. All statistical analyses, except for the Wilcoxon rank-sum test, were performed using Statistical Analysis System v9.3 (SAS Institute, Cary, NC, USA). The Wilcoxon rank-sum test in Additional file 1: Fig. 3 was performed using Seurat v3.2.3 package, as described above.

## Results

### Mutagenicity of the β-cell inducer ALK5iII used for iPIC differentiation in the Ames test

In the iPIC differentiation process, many chemical compounds are added to recapitulate differentiation signaling that occurs during human development. We evaluated the mutagenic potential of 14 compounds by excluding endogenous materials such as amino acids, sugars, and cytokines (Fig. 1a). We then analyzed these 14 compounds with two complementary and well-validated quantitative structure–activity relationship ((Q)SAR)-based bacterial mutagenicity prediction programs, Derek and CASE Ultra, both of which are commonly used for ICH M7 impurity assessment [27]. These in silico (Q)SAR-based analyses identified positive mutagenic structural alerts for five compounds: ALK5iII, LDN-193189, PD-166866, R428, and TR06141363 (Fig. 1b). Next, to examine whether these structural alerts were associated with mutagenic responses, we conducted the bacterial reverse mutation test (Ames test) with five strains in the absence of rat liver S9 fraction. Among the five strains, in which either frameshift mutations (TA98 and TA1537) or base pair substitutions (TA100, TA1535, and WP2*uvr*A) were detected in the Ames test [28], ALK5iII showed a positive response only in the TA1537 strain in the absence of rat liver S9 metabolism (Fig. 1c).

ALK5iII is a commercially available ALK5 inhibitor, which is a derivative of naphthyridine (Fig. 1d). The TA1537 strain is known to be sensitive to intercalators [29], implicating a structural mutagenicity concern. To clarify whether the mutagenic potential of ALK5iII is linked to its planar structure or capacity to inhibit ALK5, we assessed a structural analog of ALK5iII and two other representative ALK5 inhibitors. Among them, the quinoxaline-derived ALK5iII analog TR04411299 did not inhibit ALK5, but showed a positive result in the Ames test, as was observed with ALK5iII (Fig. 1c, d). Two other representative ALK5 inhibitors, SB525334 and SB431542, did not have mutagenic potential (Fig. 1d). These results suggest that ALK5iII mutagenic potential is more likely attributed to the planar naphthyridine-derived structure rather than to the ability to inhibit ALK5.

### ALK5iII displays unique off-target CDK8/19 inhibitory profile among ALK5 inhibitors

**Fig. 2:**
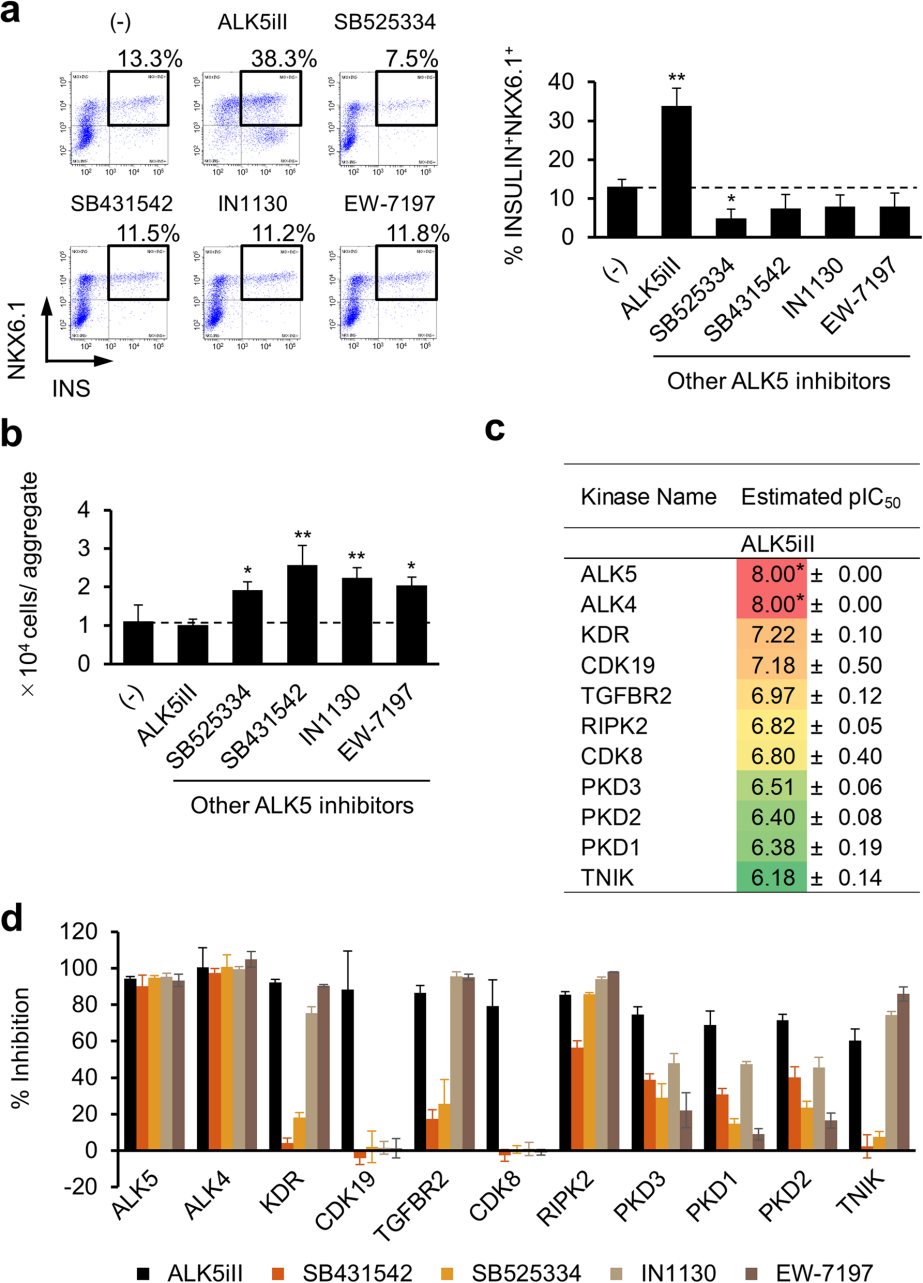
CDK8/19 are the primary candidates for the off-target action of ALK5iII during β-cell induction. **a**, **b** Representative screening results for an alternative ALK5 inhibitor to induce iPICs. Here, we selected the well-characterized ALK5 inhibitors, SB431542 and SB525334 plus randomly selected two compounds IN1130 and EW-7197, as representative compounds out of twenty. Representative dot plots (*left*, 10 μM SB525334; 10 μM SB431542; 1 μM IN1130; and 1 μM EW-7197) and average proportions of β-cells (INSULIN^+^NKX6.1^+^) (*right*) (**a**) and cell yields per aggregate (**b**). Data are shown as the mean ± SD of three independent experiments. *P < 0.05 and **P < 0.01 versus cells that were not treated by an ALK5 inhibitor; Dunnett’s test. **c** The list of kinases inhibited by ALK5iII with estimated pIC_50_ values > 6.00 in Axcelead TR-FRET-based kinase profiling assays. (ALK5iII was screened against approximately 350 kinases.) The pIC_50_ values were extrapolated from the inhibition rate at two ALK5iII concentrations, 0.1 and 1 μM. Data are shown as the mean ± SD of three independent experiments. The values “8.00*” indicate that actual pIC_50_ values were predicted to be > 8.00 from the extrapolated result. **d** Comprehensive comparison of the relative inhibition of the 11 kinases listed in **c** by ALK5iII and other representative ALK5 inhibitors, described in **a** and **b**, at a concentration of 1 μM. Data are shown as the mean ± SD from three independent experiments

To identify a non-mutagenic alternative that could replace the mutagenic ALK5iII, we screened a total of 30 ALK5 inhibitors selected from commercially available compounds and TAKEDA’s library (Additional file 1: Fig. 1a). In silico (Q)SAR-based mutagenicity prediction was applied to these 30 compounds, and 20 of them appeared to lack any structural features relevant to known mutagens (Additional file 1: Fig. 1a). We then tested whether these 20 compounds could generate iPICs enriched with β-cells. However, none of these ALK5 inhibitors promoted β-cell differentiation, marked by the expression of insulin and NKX6.1, whereas the proportion of CHGA^+^ endocrine cells was indistinguishable from that in untreated or ALK5iII inhibitor-induced cells (Fig. 2a, b; Additional file 1: Fig. 1b, c).

Because kinase inhibitors generally interact with diverse targets [30, 31], we suspected an off-target effect of ALK5iII and analyzed its kinase inhibitory profile. Inhibitory activities against 11 kinases with estimated pIC_50_ values > 6.00 have been identified for ALK5iII in tests with approximately 350 kinases (Fig. 2c). We next evaluated inhibitory activities of other representative ALK5 inhibitors, which did not promote β-cell differentiation, for the same set of 11 kinases. Interestingly, inhibitory activities toward CDK8 and CDK19 (CDK8/19) were unique to ALK5iII, whereas inhibitory properties toward other kinases, including KDR and TGFBR2, were shared by other ALK5 inhibitors (Fig. 2d). These results suggest that inhibition of CDK8/19 is of primary importance for efficient β-cell induction.

### CDK8/19 inhibition promotes efficient β-cell induction

**Fig. 3:**
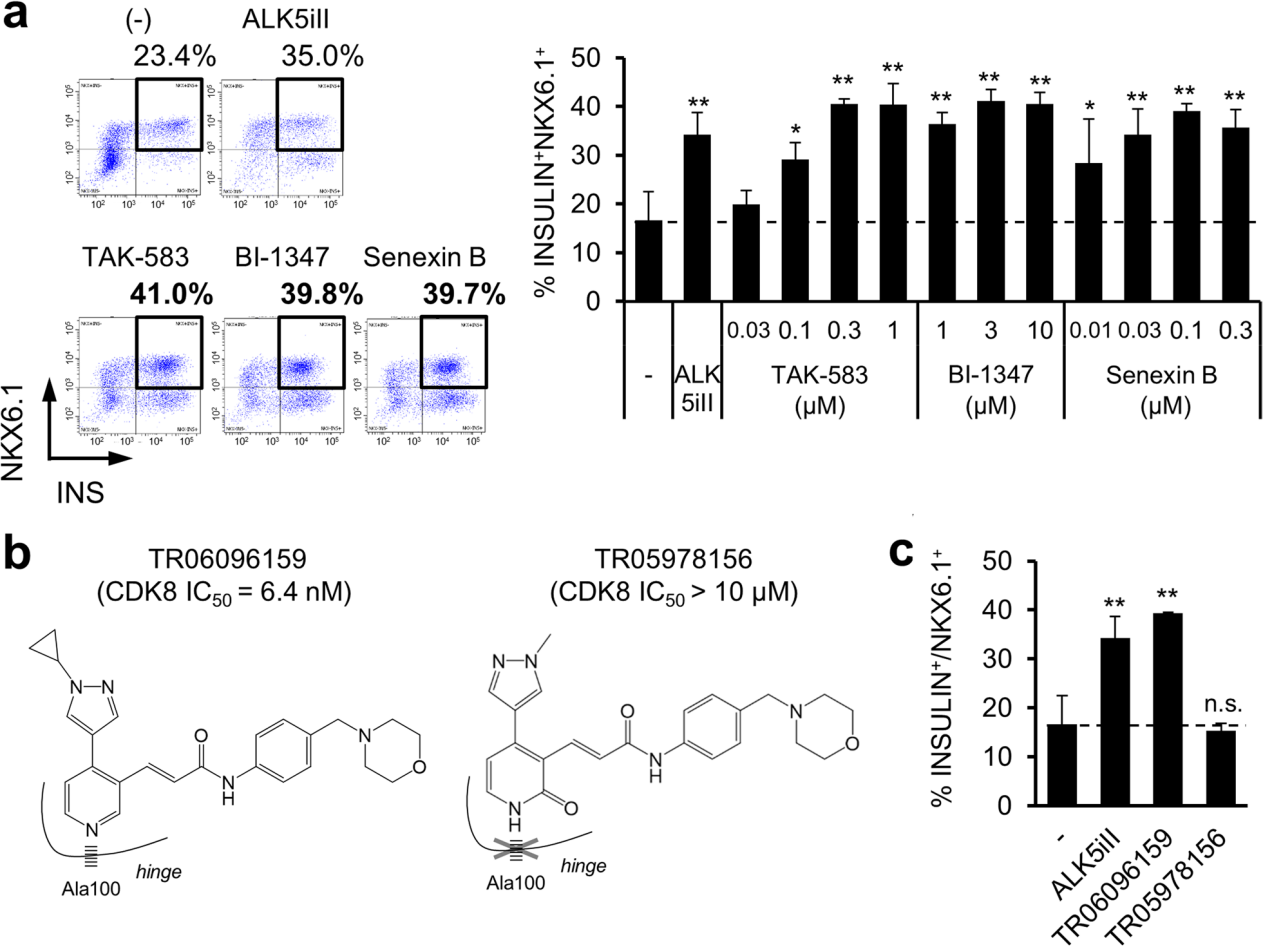
Proof of efficient β-cell induction by CDK8/19 inhibition. **a** Representative dot plots from the FCM analysis (*left,* 0.3 μM TAK-583; 3 nM BI-1347; and 0.1 μM senexin B) and dose–response relationships describing the induction of β-cells by CDK8/19 inhibitors (*right*). Data are shown as the mean ± SD of three independent experiments. *P < 0.05 and **P < 0.01 versus cells that were not treated by either ALK5iII or CDK8/19 inhibitors; Dunnett’s test. **b** Schematic docking model of a pair of on-target pharmacological tools, TR06096159 and TR05978156, bound to CDK8 (modified from the previous report [32]). **c** Proportions of β-cells differentiated with TR06096159, which binds CDK8, and TR05978156, which does not bind CDK8. Data are shown as the mean ± SD from three independent experiments. *P < 0.05 and **P < 0.01 versus untreated cells; Dunnett’s test

To elucidate whether CDK8/19 inhibition plays a pivotal role in β-cell induction, we treated cells with several CDK8/19 inhibitors, instead of ALK5iII, during endocrine cell differentiation steps from stage 5 to 7 (see the protocol scheme in Fig. 1a). All tested CDK8/19 inhibitors, that we ensured no structural concern of mutagenicity in (Q)SAR analysis in advance, shared no other kinase inhibitory activities with ALK5iII but induced β-cells efficiently in a dose-dependent manner (Fig. 3a; Additional file 1: Figs. 1d and 2a). In addition, we sought to investigate whether the inhibition of CDK8/19 was solely responsible for efficient β-cell induction by using a pair of compounds, TR06096159 and TR05978156. It was reported previously that introduction of a hydroxyl group adjacent to the hinge-binding pyridine N-position of the potent CDK8/19 inhibitor TR06096159 (CDK8 IC_50_ = 6.4 nM) drastically decreased its potency (TR05978156; CDK8 IC_50_ > 10 µM) [32]. Thus, we utilized them as an on-target pharmacological inhibition tool instead of gene silencing or knockout approaches, which are difficult to apply to cell aggregates in culture (Additional file 1: Fig. 2a; Fig. 3b). TR05978156, which cannot interact with the binding pocket of CDK8/19, did not induce β-cells efficiently, whereas TR06096159 did (Fig. 3c). These results indicate that CDK8/19 inhibition promoted efficient β-cell induction.

### Combining ALK5 inhibition and CDK8/19 inhibition mimics the effect of ALK5iII during the generation of iPICs

**Fig. 4:**
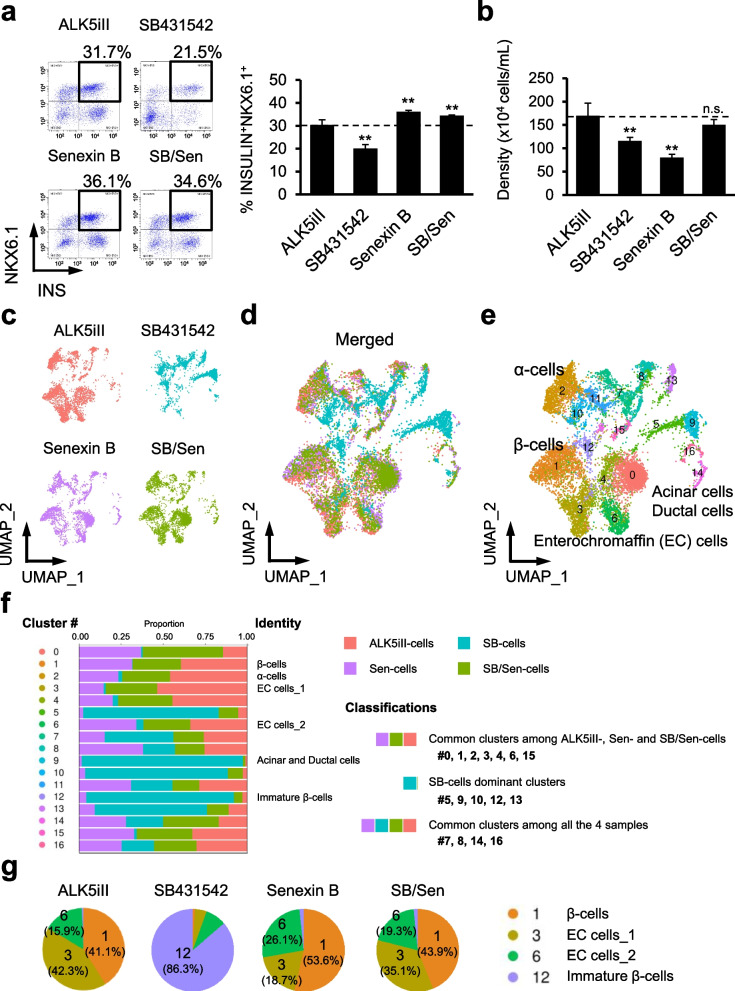

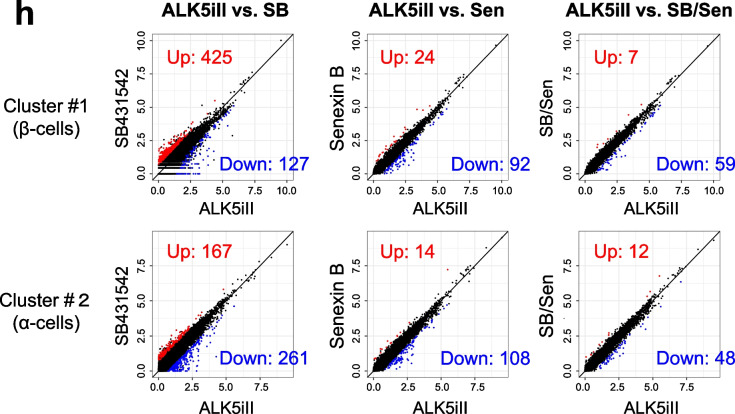
Transcriptome dissection reveals distinctive roles of ALK5 and CDK8/19 inhibition for endocrine cell induction. **a**, **b** iPICs were differentiated with 10 µM ALK5iII or another ALK5 inhibitor, 3 µM SB431542, and/or the CDK8/19 inhibitor, 0.3 µM senexin B to replace ALK5iII. Representative dot plots from FCM analysis (**a**, *left*), average proportions of β-cells (**a**, *right*), and density of obtained cells (**b**) are illustrated. Data are representative of three independent experiments and presented as the mean ± SD (*n* = 4 technical replicates). *P < 0.05 and **P < 0.01 versus data from the ALK5iII-treated cells, Dunnett’s test. **c** and **d** Individual (**c**) and merged (**d**) distributions of cells based on their gene expression profiles, shown as uniform manifold projections for four types of iPICs treated with ALK5 and CDK8/19 inhibitors as described in **a** and **b**. **e** Shared nearest neighbor clustering divided iPICs into seventeen cell clusters. Superimposed annotation indicated identified cell types based on both DEG-driven enrichment analysis and expression levels of well-validated cellular markers. **f** The proportion of cells assigned from each sample in each cluster. Total cell numbers were uniformly targeted to 3000 cells for all four samples. For “Common clusters among ALK5iII-, Sen- and SB/Sen-cells groups,” less than 5% of cells were categorized from SB-cells. For “SB-cells dominant clusters” groups, more than 66% of cells were SB-cells. **g** Internal composition of cells highly expressing *INS* and *NKX6.1* (clusters #1, 3, 6, and 12) within each sample. Pie charts describe the proportions of the cells allocated to each cluster. **h** Differentially gene expression analysis in iPIC samples compared to data from ALK5iII-treated cells. The number of up- and down-regulated genes (*P* < 0.05 and FC < 2 or >  − 2) were presented in the scatter plots in red or blue

We then aimed to obtain iPICs with a non-mutagenic β-cell inducer alternative to ALK5iII by understanding the distinct roles of ALK5 and CDK8/19 kinase inhibition. We prepared iPICs that were exposed to four types of treatment for comparison: ALK5iII (ALK5iII-cells); another ALK5 inhibitor, SB431542 (SB-cells); the CDK8/19 inhibitor senexin B (Sen-cells); and a combination of SB431542 and senexin B (SB/Sen-cells). We used these drugs at concentrations at which they caused full inhibition in the time-resolved fluorescence resonance energy transfer-based (TR-FRET) cell-free dose–response assays (Additional file 1: Fig. 2b). Notably, SB431542 and senexin B were negative in the Ames test in the absence of rat liver S9 and in (Q)SAR software prediction in silico, respectively, ensuring that these compounds are not of mutagenic concern (see the scheme in Fig. 1b, d and Additional file 1: Fig. 1a). SB/Sen-cells showed a slightly higher proportion of β-cells and comparable total cell yield with those observed in cells obtained with ALK5iII treatment (Fig. 4a, b). SB-cells had poor β-cell differentiation efficiency and decreased yield. Sen-cells showed higher β-cell differentiation efficiency, but decreased yield (Fig. 4a, b). Immunocytochemistry analysis revealed that SB-cells showed INS^+^ β-cells only in the periphery of the aggregates with porous structure, which was different from the staining pattern of ALK5iII-, Sen-, and Sen/SB-cells (Additional file 1: Fig. 3a).

To compare cellular compositions of the four types of iPICs, we performed the scRNA-seq analysis and visualized the results with dimensionality reduction using uniform manifold approximation and projection [24, 25] (Fig. 4c, d). Unsupervised cell clustering identified 17 clusters among merged cells (Fig. 4e). Expression patterns of cell identity markers, including *INS* and *GCG*, and enrichment analysis based on the publicly available database PanglaoDB (https://panglaodb.se/) [33] showed that clusters #1, 2, and 9 were close to β-, α-, and acinar/ductal cells, respectively (Fig. 4e; Additional file 1: Fig. 3c). In addition, cells in clusters #3 and 6 possessed enterochromaffin cell-specific signatures [34] (Additional file 1: Fig. 3c). Cells in cluster #12, despite high expression levels of *INS* and *NKX6.1*, were likely immature β-cells, because they did not express β-cell maturation markers, such as *MAFA* and *G6PC2*, different from cells in adjacent cluster #1 (Fig. 4e; Additional file 1: Fig. 3b, c). Of note, some β-cells might have been integrated in cluster #0 together with α-, δ-, and γ-cells, which possess adaptive cell plasticity, as highlighted by recent findings (Additional file 1: Fig. 3b, c) [35, 36]. Cells in cluster #11 displayed intermediate characteristics between hepatocytes and pancreatic α-cells, and clusters #7 and 15 most resembled neural-like cells (Fig. 4e; Additional file 1: Fig. 3b, c).

We then compared differentially abundant cell subpopulations across the four types of iPICs to understand the similarities and differences between effects of different endocrine cell inducers. Typical endocrine cell types, such as β-, α-cells, and enterochromaffin cells (cluster #0, 1, 2, 3, and 6), were almost exclusively present in ALK5iII-, Sen-, and SB/Sen-cells, whereas some other clusters (#5, 9, 10, 12, and 13) were contrastingly specific to SB-cells (Fig. 4f). Pie charts describing the subpopulations of the *INS*^+^*NKX6.1*^+^ cells (#1, 3, 6, and 12) indicate that SB/Sen-cells had comparable proportions of these subpopulations to those in ALK5iII-cells, but distinct from the distributions of the subpopulations was in Sen- and SB-cells (Fig. 4g). In addition, the number of the differentially expressed genes (DEGs) between two samples within clusters #1 and 2 (putative β- and α-cells) was the lowest in the comparison of ALK5iII-cells versus SB/Sen-cells compared to corresponding DEG numbers between other pairs (Fig. 4h). Taken together, these results suggest that SB/Sen-cells have comparable in vitro profiles to those of ALK5iII-cells.

### iPICs obtained without using ALK5iII display comparable in vivo efficacy with ALK5iII-cells in a mouse model of diabetes

**Fig. 5:**
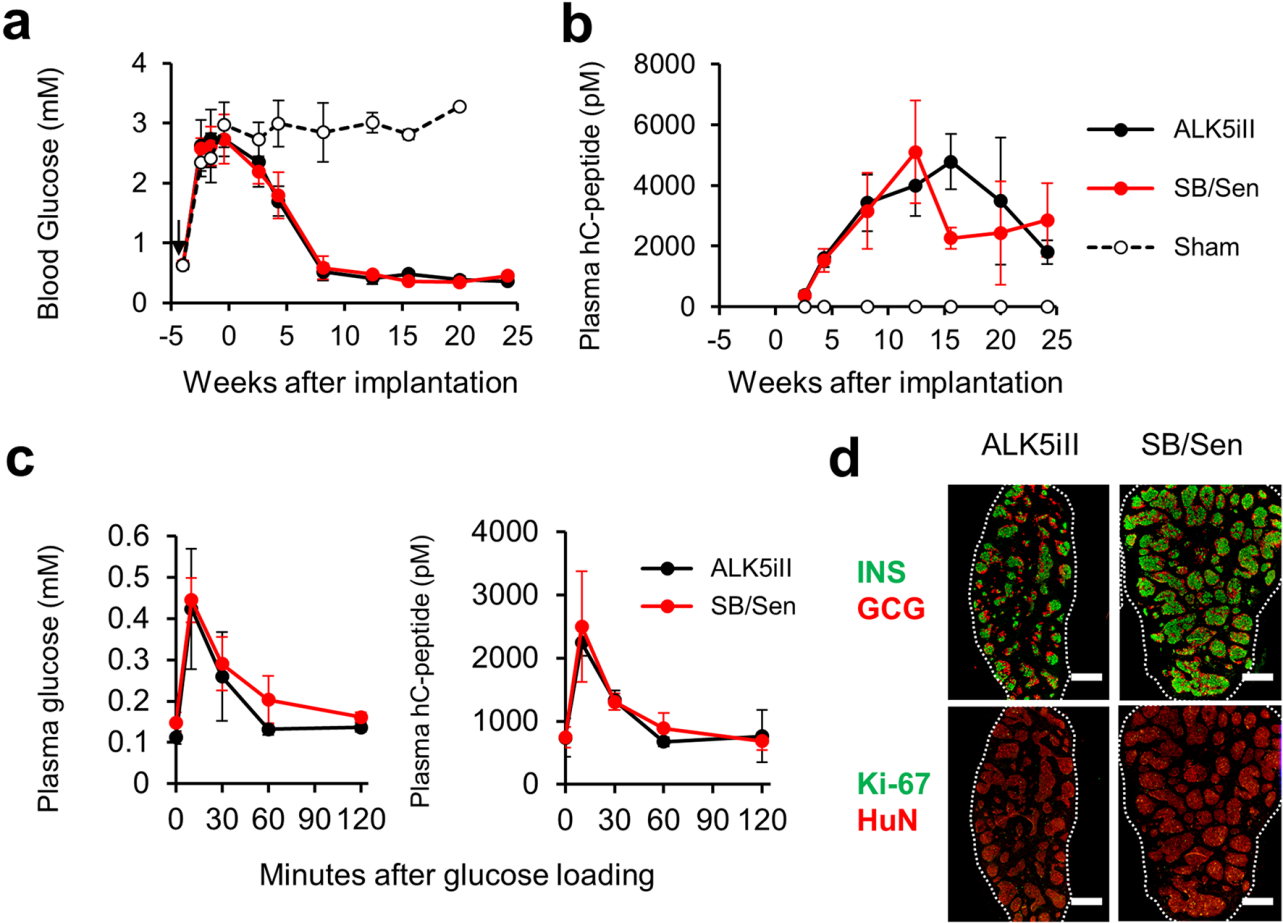
Generation of iPICs using a non-mutagenic ALK5 inhibitor and CDK8/19 inhibitor instead of mutagenic ALK5iII. **a**, **b** Blood glucose (**a**) and human C-peptide (**b**) levels after ALK5iII- and SB/Sen-cells (3 × 10^6^ cells/mouse) implantation in diabetic NOD-*scid* mice. Data are shown as the mean ± SD (n = 4–5 for ALK5iII, n = 3–4 for SB/Sen, n = 1–5 for sham). Unexpected death occurred during the period due to both hyperglycemia and hypoglycemia. All sham mice died by 21 weeks after implantation. Black arrow indicates streptozotocin injection point. **c** Plasma glucose levels during an oral glucose tolerance test at 21 weeks after implantation. Data are shown as the mean ± SD (ALK5iII-cells; n = 4, SB/Sen-cells; n = 3). **d** Representative sectional images of grafts 6 months after implantation. Sections were stained with antibodies against insulin (INS), glucagon (GCG) (green/red), and Ki-67/HuN (green/red). Scale bars (white) indicate 0.5 mm

Finally, we assessed the reproducibility of the in vivo antidiabetic efficacy of iPICs using SB/Sen-cells, which displayed in vitro profiles close to those of ALK5iII-cells (Fig. 4). When implanted into streptozotocin-induced diabetic NOD-*scid* mice, ALK5iII-cells and SB/Sen-cells showed comparable plasma glucose-lowering efficacy and human C-peptide secretion that reached normoglycemic levels within 8 weeks (Fig. 5a, b). At 21 weeks after implantation, ALK5iII- and SB/Sen-cells were subjected to an oral glucose tolerance test. Both iPICs responded to exogenous glucose challenge with a peak at 15 min (Fig. 5c). In addition, more GCG^+^ α-cells appeared in vivo, commonly between ALK5iII- and SB/Sen-cells grafts than at the time of implantation (Fig. 5d; Additional file 1: Fig. 3a). These results indicated that the therapeutic potential of iPICs could be reproduced by using a combination of non-mutagenic ALK5 inhibitors and CDK8/19 inhibitors, instead of utilizing mutagenic ALK5iII.

## Discussion

An accidental finding of the mutagenic potential of ALK5iII in the Ames test prompted us to find an alternative chemical compound for iPIC induction. However, no other ALK5 inhibitor could efficiently induce iPICs in our ALK5 inhibitor screening for alternative non-mutagenic β-cell inducers. This observation is consistent with previous reports [7, 12, 37], confirming that ALK5iII promotes differentiation from pancreatic progenitors to endocrine cells, including INS^+^ cells. Although those studies implicated the existence of an off-target effect, the actual key target remained elusive. Because kinase inhibitors are generally promiscuous and interact with many targets beyond their primary target [30, 31], we comprehensively searched for off-target kinases and identified CDK8/19 as a unique target of ALK5iII among other ALK5 inhibitors. Subsequent proof-of-mechanism studies that utilized a pair of specific CDK8/19 binder (CDK8 IC_50_ = 6.4 nM) and non-binder (CDK8 IC_50_ > 10 µM) [32] proved that CDK8/19 inhibition is essential for efficient β-cell induction, advancing our understanding of the molecular mechanisms.

The cyclin-dependent kinase CDK8 and its paralog CDK19 are components of the mediator complex and are involved in multiple downstream transcriptional processes, including activation of the TGF-β/BMP, Wnt/β-catenin, p53, serum response, and thyroid hormone-dependent pathways in multiple species and cell types [38]. CDK8 is one of the most highly expressed members of the CDK family in human islets and isolated β-cells together with CDK4 and CDK9 [39–41], but little is known about the role of CDK8 in pancreatic β-cells. A recent report demonstrated that CDK8 negatively regulates insulin secretion and represses apoptosis upon stress by silencing neuropeptide expression in mice [42]. Our data shed light on the novel role of CDK8/19 in the differentiation of β-cells in hPSC culture.

Given the previously reported pathways of CDK8/19 and β-cell differentiation, the putative mechanism of action responsible for stimulation of β-cell differentiation by CDK8/19 inhibitors is the regulation of the TGF-β/BMP and Wnt/β-catenin pathways. Activation of TGF-β receptors leads to SMAD4 phosphorylation by nuclear CDK8/19, which drives transcriptional activation and turnover by forming complexes with TGF-β receptor-regulated SMAD (R-SMAD) and coactivators [38]. In relevance to the protocol of β-cell differentiation from hPSCs, TGF-β/BMP pathways have been implicated in INS^+^ cell induction over the last decade [7, 12, 37, 43–47]. Inhibition of the TGF-β/BMP receptor by SB431542 (for ALK4/5/7) and by noggin (for BMP) at the pancreatic progenitor stage was found to promote early endocrine differentiation, but the obtained cells were still polyhormonal and immature, like fetal β-cells [46]. Indeed, ALK5 inhibition by SB431542 and others failed to promote β-cell differentiation when BMP signaling was inhibited by LDN (Fig. 2a), suggesting that only modulation of the TGF-β/BMP pathway is not enough for β-cell induction, at least for INS^+^NKX6.1^+^ cells. In contrast, in the Wnt/β-catenin pathway, nuclear CDK8/19 directly modulates β-catenin-dependent transcription as a part of the mediator complex or indirectly promotes it via inhibition of the repressive transcriptional regulator E2F1 [38]. The suppression of the Wnt/β-catenin-dependent transcription has recently been reported to affect β-cell differentiation and maturation at the endocrine cell differentiation stage in hPSC-derived cells and in mice [47–49]. Therefore, we speculate that CDK8/19 suppresses β-cell differentiation and maturation by promoting the transcription of the Wnt/β-catenin targeted genes. Taken together, the unique ability of ALK5iII to promote β-cell differentiation and maturation among ALK5 inhibitors can be attributed to CDK8/19 inhibition, possibly via the suppression of β-catenin-dependent transcription programs.

Among iPICs, Sen/SB-cells showed in vitro profile relatively close to that of ALK5iII-cells, compared with Sen-cells (Fig. 4), suggesting that inhibition of TGF-β by ALK5iII substantially affected the cell phenotype via pathways independent of the one related to CDK8/19. Activation of TGF-β receptors also affects various SMAD-independent signaling pathways, as well as SMAD- and CDK8/19-mediated transcriptions [50]. These pathways might play a substantial role in the maintenance and early endocrine differentiation in pancreatic progenitors, which possibly explains the poor cell yield in a CDK8/19 inhibitor-treated cells (Sen-cells, Fig. 4). In this study, we simply replaced ALK5iII with a combination of senexin B and SB431542 for iPIC differentiation at stages 5–7. To obtain the in vitro profile close to that of the ALK5iII-cells, it might be necessary to optimize the concentration and duration of treatment according to the activity of each pathway in the process of ALK5iII-cell generation. Nonetheless, in terms of the replacement of ALK5iII in iPIC differentiation factors for cell therapy, we reproduced the effect of ALK5iII with comparable efficacy using Sen/SB-cells. In addition, the finding that both CDK8/19 inhibitors and TGF-β inhibitors affected cell profile leads us to anticipate a possibility of fine tuning on endocrine cell differentiation with respect to the proportion of required endocrine cell types or cell maturation level. These possibilities were limited in previous differentiation protocols that used ALK5iII.

A possible limitation of this study was the unknown relationship between the mutagenic potential of the tested compounds and actual tumorigenicity of obtained cells, which were treated with the compounds during in vitro differentiation. To the best of our knowledge, there is no conclusive evidence regarding this point. For pancreatic cell differentiation, there have been no reports suggesting that the transformed cells derived from cells differentiated with ALK5iII [51, 52]. We consider that the probability of a transformation event depends on many factors, such as concentration, duration, and proliferative function of cells in the differentiation process. In the case of iPIC differentiation, the probability might be extremely low and substantially negligible, rarely observed in cell numbers regularly used in the basic research setting (*e.g.*, ~ 5 million cells per mouse [7, 52, 53]. Moreover, it might be difficult to distinguish transformed cells from undesired non-endocrine cells because cells differentiated in vitro were originally distinct from naturally developed tissues when the cells were scarce. However, the outcome might not be optimistic if the cell number were to increase dramatically, *e.g.*, during an alternative administration for islet transplantation in the clinical setting (several hundred million cells) [10]. Further investigation is needed to address these issues in the future.

## Conclusion

In terms of potential risk mitigation for future patients, however, we established that it is possible to replace mutagenic ALK5iII with non-mutagenic alternatives by discovering a true target of ALK5iII action, which was the most striking finding of this study.

## Supplementary Information

**Additional file 1**. **Supplementary Fig. 1.** Endocrine cell induction during screening for an ALK5 inhibitor and its replacement by CDK8/19 inhibitors. **Supplementary Fig. 2.** Kinase inhibition profiles of CDK8/19 inhibitors. **Supplementary Fig. 3.** Immunostaining images and enrichment analysis of ALK5iII-, Sen-, SB-, and Sen/SB-cells. **Supplementary Table 1.** List of primary antibodies used in immunofluorescence staining in this study.

## Data Availability

All data on this study are available within this publication and its supplementary information or can be provided upon request. Gene expression data are available at GEO, accession number GSE215152.

## Abbreviations

ALK5iII: Activin receptor-like kinase 5 inhibitor II
CDK8/19: Cyclin-dependent kinases 8 and 19
DEGs: Differentially expressed genes
hiPSCs: Human-induced pluripotent stem cells
hPSCs: Human pluripotent stem cells
iPIC: HiPSC-derived pancreatic islet-like cells
(Q)SAR: Quantitative structure–activity relationship
R-SMAD: TGF-β receptor-regulated SMAD
TGF-β: Transforming growth factor-β
TR-FRET: Time-resolved fluorescence resonance energy transfer

## References

[1] American DA (2021). Standards of medical care in diabetes-2021 abridged for primary care providers. Clin Diabetes.

[2] Bellin MD, Dunn TB (2020). Transplant strategies for type 1 diabetes: whole pancreas, islet and porcine beta cell therapies. Diabetologia.

[3] Vantyghem MC, de Koning EJP, Pattou F, Rickels MR (2019). Advances in beta-cell replacement therapy for the treatment of type 1 diabetes. Lancet.

[4] de Klerk E, Hebrok M (2021). Stem cell-based clinical trials for diabetes mellitus. Front Endocrinol (Lausanne).

[5] Toyoda T, Mae S, Tanaka H, Kondo Y, Funato M, Hosokawa Y (2015). Cell aggregation optimizes the differentiation of human ESCs and iPSCs into pancreatic bud-like progenitor cells. Stem Cell Res.

[6] Russ HA, Parent AV, Ringler JJ, Hennings TG, Nair GG, Shveygert M (2015). Controlled induction of human pancreatic progenitors produces functional beta-like cells in vitro. EMBO J.

[7] Rezania A, Bruin JE, Arora P, Rubin A, Batushansky I, Asadi A (2014). Reversal of diabetes with insulin-producing cells derived in vitro from human pluripotent stem cells. Nat Biotechnol.

[8] Pagliuca FW, Millman JR, Gurtler M, Segel M, Van Dervort A, Ryu JH (2014). Generation of functional human pancreatic beta cells in vitro. Cell.

[9] Nair GG, Liu JS, Russ HA, Tran S, Saxton MS, Chen R (2019). Recapitulating endocrine cell clustering in culture promotes maturation of human stem-cell-derived beta cells. Nat Cell Biol.

[10] Ramzy A, Thompson DM, Ward-Hartstonge KA, Ivison S, Cook L, Garcia RV (2021). Implanted pluripotent stem-cell-derived pancreatic endoderm cells secrete glucose-responsive C-peptide in patients with type 1 diabetes. Cell Stem Cell.

[11] Ma X, Zhu S (2017). Chemical strategies for pancreatic beta cell differentiation, reprogramming, and regeneration. Acta Biochim Biophys Sin (Shanghai).

[12] Rezania A, Riedel MJ, Wideman RD, Karanu F, Ao Z, Warnock GL (2011). Production of functional glucagon-secreting alpha-cells from human embryonic stem cells. Diabetes.

[13] Hong SG, Dunbar CE, Winkler T (2013). Assessing the risks of genotoxicity in the therapeutic development of induced pluripotent stem cells. Mol Ther.

[14] Simonatto M, Latella L, Puri PL (2007). DNA damage and cellular differentiation: more questions than responses. J Cell Physiol.

[15] Mochida T, Ueno H, Tsubooka-Yamazoe N, Hiyoshi H, Ito R, Matsumoto H (2020). Insulin-deficient diabetic condition upregulates the insulin-secreting capacity of human induced pluripotent stem cell-derived pancreatic endocrine progenitor cells after implantation in mice. Diabetes.

[16] Hiyoshi H, Sakuma K, Tsubooka-Yamazoe N, Asano S, Mochida T, Yamaura J (2022). Characterization and reduction of non-endocrine cells accompanying islet-like endocrine cells differentiated from human iPSC. Sci Rep.

[17] Hu W, Qiu B, Guan W, Wang Q, Wang M, Li W (2015). Direct conversion of normal and Alzheimer's disease human fibroblasts into neuronal cells by small molecules. Cell Stem Cell.

[18] Yamamoto K, Kishida T, Nakai K, Sato Y, Kotani SI, Nishizawa Y (2018). Direct phenotypic conversion of human fibroblasts into functional osteoblasts triggered by a blockade of the transforming growth factor-beta signal. Sci Rep.

[19] Nakagawa M, Taniguchi Y, Senda S, Takizawa N, Ichisaka T, Asano K (2014). A novel efficient feeder-free culture system for the derivation of human induced pluripotent stem cells. Sci Rep.

[20] Suenaga R, Konagaya S, Yamaura J, Ito R, Tanaka S, Ishizaki Y (2022). Microwell bag culture for large-scale production of homogeneous islet-like clusters. Sci Rep.

[21] Hirozane Y, Toyofuku M, Yogo T, Tanaka Y, Sameshima T, Miyahisa I (2019). Structure-based rational design of staurosporine-based fluorescent probe with broad-ranging kinase affinity for kinase panel application. Bioorg Med Chem Lett.

[22] Stuart T, Butler A, Hoffman P, Hafemeister C, Papalexi E, Mauck WM (2019). Comprehensive Integration of Single-Cell Data. Cell.

[23] Butler A, Hoffman P, Smibert P, Papalexi E, Satija R (2018). Integrating single-cell transcriptomic data across different conditions, technologies, and species. Nat Biotechnol.

[24] McInnes LHJ. UMAP: uniform manifold approximation and projection for dimension reduction. https://arxiv.org/abs/180203426. 2018.

[25] McInnes L, Healy J, Saul N, Groberger L (2018). UMAP: uniform manifold approximation and projection. J Open Source Softw.

[26] Yu G, Wang LG, Han Y, He QY (2012). clusterProfiler: an R package for comparing biological themes among gene clusters. OMICS.

[27] Jayasekara PS, Skanchy SK, Kim MT, Kumaran G, Mugabe BE, Woodard LE (2021). Assessing the impact of expert knowledge on ICH M7 (Q)SAR predictions. Is expert review still needed?. Regul Toxicol Pharmacol.

[28] OECD. *Test no. 471: bacterial reverse mutation test,* OECD Guidelines for the Testing of Chemicals, Section 4. OECD Publishing, Paris. 2020.

[29] Hamel A, Roy M, Proudlock R. Chapter 4—the bacterial reverse mutation test. Genetic Toxicology Testing. 2016:79–138.

[30] Gregori-Puigjane E, Setola V, Hert J, Crews BA, Irwin JJ, Lounkine E (2012). Identifying mechanism-of-action targets for drugs and probes. Proc Natl Acad Sci USA.

[31] Eberl HC, Werner T, Reinhard FB, Lehmann S, Thomson D, Chen P (2019). Chemical proteomics reveals target selectivity of clinical Jak inhibitors in human primary cells. Sci Rep.

[32] Fujimoto J, Hirayama T, Hirata Y, Hikichi Y, Murai S, Hasegawa M (2017). Studies of CDK 8/19 inhibitors: Discovery of novel and selective CDK8/19 dual inhibitors and elimination of their CYP3A4 time-dependent inhibition potential. Bioorg Med Chem.

[33] Franzen O, Gan LM, Bjorkegren JLM. PanglaoDB: a web server for exploration of mouse and human single-cell RNA sequencing data. Database (Oxford). 2019;2019. 10.1093/database/baz046 PMC6450036 30951143

[34] Veres A, Faust AL, Bushnell HL, Engquist EN, Kenty JH, Harb G (2019). Charting cellular identity during human in vitro beta-cell differentiation. Nature.

[35] Perez-Frances M, van Gurp L, Abate MV, Cigliola V, Furuyama K, Bru-Tari E (2021). Pancreatic Ppy-expressing gamma-cells display mixed phenotypic traits and the adaptive plasticity to engage insulin production. Nat Commun.

[36] Thorel F, Nepote V, Avril I, Kohno K, Desgraz R, Chera S (2010). Conversion of adult pancreatic alpha-cells to beta-cells after extreme beta-cell loss. Nature.

[37] Kunisada Y, Tsubooka-Yamazoe N, Shoji M, Hosoya M (2012). Small molecules induce efficient differentiation into insulin-producing cells from human induced pluripotent stem cells. Stem Cell Res.

[38] Galbraith MD, Donner AJ, Espinosa JM (2010). CDK8: a positive regulator of transcription. Transcription.

[39] Taneera J, Fadista J, Ahlqvist E, Zhang M, Wierup N, Renstrom E (2013). Expression profiling of cell cycle genes in human pancreatic islets with and without type 2 diabetes. Mol Cell Endocrinol.

[40] Nica AC, Ongen H, Irminger JC, Bosco D, Berney T, Antonarakis SE (2013). Cell-type, allelic, and genetic signatures in the human pancreatic beta cell transcriptome. Genome Res.

[41] Blodgett DM, Nowosielska A, Afik S, Pechhold S, Cura AJ, Kennedy NJ (2015). Novel observations from next-generation RNA sequencing of highly purified human adult and fetal islet cell subsets. Diabetes.

[42] Xue J, Scotti E, Stoffel M (2019). CDK8 regulates insulin secretion and mediates postnatal and stress-induced expression of neuropeptides in pancreatic beta cells. Cell Rep.

[43] D'Amour KA, Bang AG, Eliazer S, Kelly OG, Agulnick AD, Smart NG (2006). Production of pancreatic hormone-expressing endocrine cells from human embryonic stem cells. Nat Biotechnol.

[44] Kroon E, Martinson LA, Kadoya K, Bang AG, Kelly OG, Eliazer S (2008). Pancreatic endoderm derived from human embryonic stem cells generates glucose-responsive insulin-secreting cells in vivo. Nat Biotechnol.

[45] Schulz TC, Young HY, Agulnick AD, Babin MJ, Baetge EE, Bang AG (2012). A scalable system for production of functional pancreatic progenitors from human embryonic stem cells. PLoS ONE.

[46] Nostro MC, Sarangi F, Ogawa S, Holtzinger A, Corneo B, Li X (2011). Stage-specific signaling through TGFbeta family members and WNT regulates patterning and pancreatic specification of human pluripotent stem cells. Development.

[47] Sharon N, Vanderhooft J, Straubhaar J, Mueller J, Chawla R, Zhou Q (2019). Wnt signaling separates the progenitor and endocrine compartments during pancreas development. Cell Rep.

[48] Vethe H, Bjorlykke Y, Ghila LM, Paulo JA, Scholz H, Gygi SP (2017). Probing the missing mature beta-cell proteomic landscape in differentiating patient iPSC-derived cells. Sci Rep.

[49] Vethe H, Ghila L, Berle M, Hoareau L, Haaland OA, Scholz H (2019). The effect of Wnt pathway modulators on human iPSC-derived pancreatic beta cell maturation. Front Endocrinol (Lausanne).

[50] Derynck R, Zhang YE (2003). Smad-dependent and Smad-independent pathways in TGF-beta family signalling. Nature.

[51] Maxwell KG, Millman JR (2021). Applications of iPSC-derived beta cells from patients with diabetes. Cell Rep Med.

[52] Hogrebe NJ, Augsornworawat P, Maxwell KG, Velazco-Cruz L, Millman JR (2020). Targeting the cytoskeleton to direct pancreatic differentiation of human pluripotent stem cells. Nat Biotechnol.

[53] Robert T, De Mesmaeker I, Stange GM, Suenens KG, Ling Z, Kroon EJ (2018). Functional beta cell mass from device-encapsulated hESC-derived pancreatic endoderm achieving metabolic control. Stem Cell Rep.

