# Supplementary material for: CDK8/19 inhibition plays an important role in pancreatic β-cell induction from human iPSCs
Source: Stem Cell Res Ther. 2023 Jan 5;14:1. doi: 10.1186/s13287-022-03220-4 (PMC9814340; doi:10.1186/s13287-022-03220-4)
Supplement: Supplementary file 1 — Additional file 1. Supplementary Fig. 1. Endocrine cell induction during screening for an ALK5 inhibitor and its replacement by CDK8/19 inhibitors. Supplementary Fig. 2. Kinase inhibition profiles of CDK8/19 inhibitors. Supplementary Fig. 3. Immunostaining images and enrichment analysis of ALK5iII-, Sen-, SB-, and Sen/SB-cells. Supplementary Table 1. List of primary antibodies used in immunofluorescence staining in this study. [file 13287_2022_3220_MOESM1_ESM.docx]

**Supplementary Information for**

CDK8/19 inhibition plays an important role in pancreatic β-cell induction from human iPSCs

Kensuke Sakuma^1,2*^, Noriko Tsubooka-Yamazoe^1,2^, Kiyohiro Hashimoto^3^, Nozomu Sakai^4^, Shinya Asano^5^, Saori Watanabe-Matsumoto^2,7^, Takeshi Watanabe^3^, Bunnai Saito^4^, Hirokazu Matsumoto^2,6^, Hikaru Ueno^1,2^, Ryo Ito^1,2^, and Taro Toyoda^2,7*^

^1^iPSC-derived Pancreatic Islet Cell (iPIC) Therapy Department, Orizuru Therapeutics Inc., Fujisawa, Kanagawa 251-8555, Japan; ^2^Takeda-CiRA Joint Program for iPS Cell Applications (T-CiRA), Fujisawa, Kanagawa 251-8555, Japan; ^3^Drug Safety Research and Evaluation Group, Takeda Pharmaceutical Company Limited, Kanagawa 251-8555, Japan; ^4^Drug Discovery Sciences, Takeda Pharmaceutical Company Limited, Kanagawa 251-8555, Japan; ^5^Integrated & Translational Science, Axcelead Drug Discovery Partners, Inc., Fujisawa, Kanagawa 251-8555, Japan; ^6^T-CiRA Discovery and Innovation, Takeda Pharmaceutical Company Limited, Kanagawa 251-8555, Japan; ^7^Department of Life Science Frontiers, Center for iPS Cell Research and Application (CiRA), Kyoto University, Kyoto 606-8507, Japan

*To whom correspondence may be addressed.

**Email:**   [kensuke.sakuma@orizuru-therapeutics.com;](mailto:%20kensuke.sakuma@orizuru-therapeutics.com;) [t.toyoda@cira.kyoto-u.ac.jp](mailto:t.toyoda@cira.kyoto-u.ac.jp)

**This PDF file includes:**

Supplementary text

Supplementary Figures 1–3

Supplementary Table 1

**Supplementary Fig. 1. Endocrine cell induction during screening for an ALK5 inhibitor and its replacement by CDK8/19 inhibitors.**

**a** Flow chart of candidate compound selection during screening for alternative non-mutagenic ALK5 inhibitors to induce iPICs.

**b** Dose-response preliminary studies during ALK5 inhibitor screening to determine the concentrations of alternative ALK5 inhibitors in Fig. 2a, b and Supplementary Fig. 1c. Bar graphs in black and line graphs in red indicate the proportions of β-cells (INSULIN^+^NKX6.1^+^) (*left*) and the yielded cell numbers (*right*), respectively.

**c** Representative dot plots from the FCM analysis and mean proportions of endocrine cells (CHGA^+^) during ALK5 inhibitor screening, as in Fig. 2a, b. Data are shown as the mean ± SD of three independent experiments. n.s.; not significant versus values in cells without ALK5iII treatment, Dunnett's test.

**d** Representative dot plots from the FCM analysis and mean proportions of endocrine cells (CHGA^+^) during experiments on the replacement of ALK5iII by CDK8/19 inhibitors, corresponding to Fig. 3a. Data are shown as the mean ± SD of three independent experiments. n.s., not significant versus values in cells without ALK5iII treatment, Dunnett's test.

**Supplementary Fig. 2. Kinase inhibition profiles of CDK8/19 inhibitors.**

**a** Comprehensive comparison of the relative inhibition of the 11 kinases listed in Fig. 2c by CDK8/19 inhibitors, described in Fig. 3, at a concentration of 1 μM. Data are shown as the mean ± SD of three independent experiments.

**b** Dose-response curves of the TR-FRET competitive binding assay describing binding of ALK5iII, another ALK5 inhibitor (SB431542), and CDK8/19 inhibitors (TAK-583, BI-1347, and senexin B) to ALK5 and CDK8/19, respectively.

**Supplementary Fig. 3. Immunostaining images and enrichment analysis of ALK5iII-, Sen-, SB-, and Sen/SB-cells.**

**a** Representative images of cells cryosectioned and stained for insulin (INS)/glucagon (GCG) (green/red) and nuclei (blue). Scale bars (white) indicate 0.1 mm.

**b** Cell type enrichment analysis of cell type marker from PanglaoDB using differentially expressed genes, which were defined as greater than 1.5-fold change at the statistical significance of *P <* 0.05, as determined by the Wilcoxon rank sum test.

**c** Single-cell expression of representative cluster-determinant markers in iPICs.

**Supplementary Table 1. List of primary antibodies used in immunofluorescence staining in this study.**
